# Antiangiogenesis and antioxidant activity of ethanol extracts of *Pithecellobium jiringa*

**DOI:** 10.1186/1472-6882-12-210

**Published:** 2012-11-05

**Authors:** Nahdzatul Syima Muslim, Zeyad D Nassar, Abdalrahim FA Aisha, Armaghan Shafaei, Norshirin Idris, AminMalikShahAbdul Majid, Zhari Ismail

**Affiliations:** 1 EMAN Testing & Research Laboratory, Department of Pharmacology, School of Pharmaceutical Sciences, Universiti Sains Malaysia, Minden, Penang 11800, Malaysia; 2 School of Pharmacy, University of Queensland, Brisbane, QLD, Australia; 3 Department of Pharmaceutical Chemistry, School of Pharmaceutical Sciences, Universiti Sains Malaysia, Minden, Penang 11800, Malaysia

**Keywords:** Pithecellobium jiringa, Antiangiogenesis, Antioxidant, Phytochemical analysis

## Abstract

**Background:**

Angiogenesis plays a critical role in embryonic development and various physiological processes. However, excessive angiogenesis is associated with several pathological conditions including cancer. *Pithecellobium jiringa* (Jack) Prain is a traditional medicinal plant from the family Leguminosae. It is native to the Southeast Asia, where it has been used traditionally for treatment of various ailments such as hypertension and diabetes. The present work is aimed to study antioxidant and antiangiogenesis activities of *P. jiringa* ethanol extracts.

**Methods:**

*P. jiringa* fruit rinds were extracted with ethanol and 50% ethanol. The antioxidant property was analysed using, 1,1-diphenyl-2-picryl-hydrazyl free radical scavenging assay. Phytochemical analysis was performed using thin layer chromatography and colorimetric methods. Then, cell growth inhibition was studied against a panel of human cell lines by MTT test. *In vitro* inhibition of angiogenesis was studied by the following assays: isolated rat aortic rings cell viability, colony formation, endothelial cell migration, endothelial tube formation on matrigel, and expression of vascular endothelial growth factor by endothelial cells. *In vivo* antiangiogenesis effect was studied by utilising fertilised chick embryos assay. The results were statistically analysed by analysis of variance.

**Results:**

Ethanolic and 50% hydro-ethanolic extracts showed relatively high concentration of total phenolics associated with potent antioxidant activity. The rat aortic rings study conducted showed potent inhibition of the microvessels outgrowth with IC_50s_ 5.27 ± 0.81 μg/ml (ethanolic) and 4.45 ± 0.63 μg/ml (50% hydro-ethanolic). Both extracts arrested the growth of human endothelial cells via down-regulation of VEGF expression, leading to inhibition of other angiogenesis cascades including migration of endothelial cells, and formation of capillary network on matrigel matrix. The extracts also inhibited the neovascularisation of chick embryo chorioallantoic membrane.

**Conclusions:**

*P. jiringa* extracts inhibit angiogenesis by blocking the VEGF expression thus inhibiting endothelial cells proliferation, migration and differentiation most likely due to presence of the antioxidant phenolics.

## Background

Angiogenesis is the formation of new blood vessels sprouting from existing vascularisation [1]. The process plays essential role in embryonic development, wound healing and reproductive functions. In early 1970s, it was noted that solid tumours appear to be highly vascularised [2]. It was then established that all solid tumours are dependent on the angiogenesis in order for the tumour to grow larger and metastasize [1,2]. Endothelial cells in tumour bed tend to be more susceptible to cytotoxic agents due to their high proliferation rate. In addition, endothelial cells, on the contrary to cancerous cells, are genetically stable as they do not undergo mutations and hence more sensitive to apoptotic effects of the cytotoxic agents. Thus, these features of endothelial cells make them a compelling target for antiangiogenesis treatment [3]. Consequently, cytotoxic agents pose as candidates as antiangiogenic agents on top of their potent activity in causing death of cancerous cells. Paclitaxel, an anti-cancer drug has been shown to inhibit proliferation of endothelial cells as well as the cells’ migration and invasiveness dose-dependently both *in vitro* and *in vivo*[3].

Extensive studies have been conducted to assess the role of oxidative stress and hence the use of antioxidants in the prevention of many diseases such as cancer, inflammation, and atherosclerosis [4-6]. In the initial stage of these diseases, oxidative stress plays a major role in damaging essential components of cells [4]. To date, there have been extensive studies on natural product compounds and extracts that showed potent antiangiogenic activity, in conjunction to having good antioxidant activities [7-10].

*Pithecellobium jiringa* (Jack) Prain is a traditional medicinal plant native to the Southeast Asia which belongs to the family Leguminosae [11]. Traditionally, a drink mixture of pounded whole fruit with ginger is taken daily to eliminate bladder stones [12]. The plant has been traditionally used in treating hypertension and diabetes, the fruit rinds are also used for flavouring and as fragrance in manufacturing traditional soaps, shampoos and detergents. The matured leaves of the plant are burnt to ashes as a cure for itchy parts of the body [13,14]. Though there is a widespread traditional use of the whole fruit as anti-diabetes agent, *P. jiringa* has not been studied extensively [14]. This study has been undertaken in order to investigate the antioxidant and antiangiogenic activity of *Pithecellobium jiringa* ethanolic and 50% hydro-ethanolic extracts.

## Methods

### Materials

Analytical grade solvents were purchased from Avantor Performance Materials (Petaling Jaya, Selangor, Malaysia). Earles’ salt (M199) medium, trypsin, Dulbecco’s modified eagle medium (DMEM), minimum essential medium (MEM), fibrinogen and foetal bovine serum were obtained from Bio-Diagnostics (Petaling Jaya, Selangor, Malaysia). Aprotinin, L-glutamine, thrombin, sodium chloride, fungizone, gentamycin, 6-aminocaproic acid, suramin, dimethyl sulfoxide (DMSO), crystal violet, phosphate buffered saline (PBS), 3-(4,5-dimethylthiazol-2-yl-2,5-diphenyl) tetrazolium bromide (MTT), paraformaldehyde, agarose, potassium acetate, aluminum chloride, 1,1-diphenyl-2-picryl-hydrazyl (DPPH), quercetin, gallic acid, betulinic acid, Folin-Ciocalteau reagent, sodium carbonate, anisaldehyde reagent, natural product-polyethylene glycol 4000’ reagent (NP-PEG) and Dragendorff reagent were from Sigma-Aldrich (Subang Jaya, Selangor, Malaysia). Human umbilical vein endothelial cells (HUVEC), hormone-dependent breast carcinoma cells (MCF 7), human hepatocarcinoma cells (Hep G2) and normal colonic fibroblasts (CCD-18Co) were purchased from ATCC (Manassas, Virginia). Endothelial cell medium (ECM) was obtained from Team Medical Scientific (Shah Alam, Selangor, Malaysia). Matrigel matrix was purchased from Bio-Diagnostics (Petaling Jaya, Selangor, Malaysia), and VEGF-165 ELISA kit was obtained from Chemtron Biotechnology Sdn Bhd (Kuala Lumpur, Malaysia). All other chemicals used in the study were of analytical grade.

### Plant material and extraction

The plant material was authenticated by the Herbarium of School of Biological Sciences, Universiti Sains Malaysia, where a voucher specimen was deposited (Reference number of 11242). The fruit rinds were separated from the seeds and oven-dried at 40°C and powdered using a milling machine (Retsch GmbH, Germany). Fine powdered material (30 g) was macerated separately in 500 ml ethanol (EtOH) and 50% ethanol (EW) overnight at 40°C with intermittent shaking. After cooling, extracts were filtered using Whatman filter paper No.1 (Whatman, England), concentrated at 50°C under vacuum using a rotary evaporator (RE121 Buchi, Switzerland), and freeze-dried using freeze-drying system (Labconco, USA).

### Experimental animals

The thoracic aortas were excised from 8 – 12 weeks old male Sprague Dawley rats obtained from the Animal House Facility, Universiti Sains Malaysia. The rats were kept in well ventilated cages and allowed to free access of tap water and normal laboratory diet. All experimental procedures were executed following the Animal Ethics Guidelines of Universiti Sains Malaysia (USM/Animal Ethics Approval/2011/(66)(302)).

### Rat aortic ring assay

The assay was performed as previously described [15]. Briefly, aortic rings of about 1 mm thickness excised from thoracic aortas were placed into a 48-wells plate filled with 300 μl of serum-free M199 medium supplemented with 3 mg/ml fibrinogen, 5 μg/ml aprotinin and 1% L-glutamine. Thrombin (10 μl at 50 NIH U/ml in 0.15 M NaCl) was then added and incubated for 90 min at 37°C. Subsequently, various concentrations of the test extracts were mixed with a second layer made of 300 μl M199 medium and containing 1% L-glutamine, 2.5 μg/ml fungizone, 60 μg/ml gentamycin, 0.1% 6-aminocaproic acid and 20% heat inactivated foetal bovine serum (HIFBS), and were added into each well. Suramin and 1% DMSO were used as positive and negative control respectively. The plate was incubated at 37°C in a humidified CO_2_ incubator. The second layer was replaced with a freshly prepared one containing the respective tested extracts on day 4. On day 5, the explants were visualised and photographed using an inverted light microscope (Olympus) at 4× magnification. The blood vessels outgrowth of an explant was quantified by using Leica QWin computerised imaging software [16].

### Cell viability assay

Inhibition of cell growth was evaluated by the MTT test. The cells were treated with extracts as well as 1% DMSO as a negative control for 48 h. Cell viability was then assessed following a previously described method [17]. The absorbance of the treated cells was then taken by using a microtitre plate reader (Tecan, Austria) at 570 nm. The results are presented as average percentage of cell viability to that of the negative control.

### Colony formation assay

The colony formation assay was conducted following a previously described method [18]. Single cell suspension of HUVEC cells was seeded at 500 cells/ml in a 6-well plate and incubated for 24 h to allow attachment. Subsequently, culture medium containing various concentrations of the test extracts was added; betulinic acid at 20 μg/ml was used as a positive control and 1% DMSO served as a negative control. After a 48 h treatment, the medium was discarded, the cells were washed by PBS, and a fresh medium was added. On the sixth day of incubation, the cells were fixed with 4% (v/v) paraformaldehyde and subsequently stained with 0.2% (w/v) crystal violet solution in PBS. Colonies consisting of more than 50 cells were counted by using a stereomicroscope, and the results are reported as a mean percentage of survival fraction ± SD (n=2).

### Cell migration assay

Cell migration was performed following previously described procedures [19]. In brief, HUVEC cells were seeded in a 6-well plate until a confluent monolayer was formed. Subsequently, a wound was created using a sterile 200 μl micropipette tip. The detached cells were removed by washing with PBS and the plates were then treated with the test extracts at 0.5 μg/ml. The wounds were photographed after 12 and 18 h, and the width of the wound was measured using Leica QWin imaging system; 10 fields per well were taken and a minimum of 30 readings per field were measured. The results are presented as a mean percentage of migration inhibition in comparison to control ± SD (n=3).

### Tube formation assay on matrigel matrix

The matrigel matrix (5 mg/ml) was obtained by diluting the stock at 1:1 with serum-free medium, before 300 μl of the solution was transferred into each well of a 48-well plate. The matrix was allowed to solidify at 37°C and 5% CO_2_ for 45 min. The HUVEC cells were then trypsinized and seeded (3 × 10^4^cells/well) in 100 μl fresh medium containing various concentrations of the test extracts. After 6 h incubation, the tubular structures were visualised and photographed under an inverted light microscope at 4× magnification (Olympus, Japan). Quantitative assessment of the tube formation inhibition was carried out by using the ImageJ Software by measuring the area of the tubular structures [20]. The results are presented as a mean percentage inhibition ± SD (n=3).

### VEGF concentration in HUVEC cells lysates

The VEGF concentration was determined by using a human VEGF-165 ELISA kit (Raybio, USA) following the manufacturer’s protocols. HUVEC cells were treated with the test extracts at 2.5 μg/ml for 24 h before the cell lysates were prepared using cell lysis buffer provided with the kit. A calibration curve of VEGF standard was prepared at the same time. Subsequently, the concentration of VEGF in cell lysates was quantified using the log-log regression equation of the standard calibration curve (y = 0.0099x^0.6137^, *R*^2^ = 0.996). The experiment was repeated two times in triplicates.

### *In vivo* chorioallantoic membrane assay

The *in vivo* antiangiogenic effect of the test extracts was investigated by CAM assay as described previously [21]. Fertilized chicken eggs of 5-days old were obtained from a local hatchery. Albumin (5 ml) was withdrawn and the eggs were incubated horizontally to allow the CAM detachment of the shell. The extracts were dissolved in ethanol and prepared as agarose discs of 1.2% at extracts concentration of 25 and 50 μg/disc. Discs contained the vehicle only (ethanol) were used as negative control. A small window was made on the shell through which the discs were applied to the CAM. The window was closed back and sealed with a sterile surgical tape and the eggs were incubated for another 24 h. The images of each treated CAM were captured under dissecting microscope. The blood vessels in the disc application site were counted to calculate the percentage inhibition [22].

### DPPH scavenging activity

The free radical scavenging activity of the test extracts was assessed by 1,1-diphenyl-2-picryl-hydrazyl (DPPH) [23]. The assay was done in a 96-well microplate. Briefly, 100 μl of extracts (1.5625 - 100 μg/ml) and gallic acid as the standard (0.3125 - 20 μg/ml) prepared in methanol were loaded into the wells. Then, 100 μl of 200 μM DPPH solution in methanol was added. After 30 min of incubation at room temperature (RT), the absorbance was measured at 517 nm using a microplate reader (Tecan, Austria) against methanol as a blank. The negative control was made of 100 μl methanol and 100 μl of 200 μM DPPH solution. Reduced optical density indicated higher free radical scavenging activity. The free radical scavenging activity of the extracts and gallic acid was determined as follows:

Percentage of free radical scavenging activity = [1 − (A_S_ − A_B_)/A_C_ − AB] × 100, where:

AS: absorbance of sample/standard

AB: absorbance of blank

AC: absorbance of control

### Phytochemical screening

*P. jiringa* fruit rinds extracts were screened for presence of various classes of chemical constituents as previously described [24]. High performance thin layer chromatography (HPTLC) (Camag, Switzerland) was conducted using a mobile phase system of ethyl acetate-formic acid-acetic acid-water (100:11:11:26). The developed TLC plates were then sprayed with various spraying reagents as the following; anisaldehyde reagent for terpenoids, natural product reagent for flavonoids, Dragendorff reagent for alkaloids and Folin-Ciocalteu reagent for phenolic compounds. After spraying, the plates were heated at 100°C for 5 min, and were visualised at 366 nm. The presence of the compounds was confirmed by the presence of bands after spraying with respective reagents. All tests were repeated thrice.

### Estimation of total phenolics content

The total phenolics content was determined using a colorimetric assay [25]. Test extracts (100 μl at 1 mg/ml in methanol) was added to 750 μl Folin-Ciocalteau reagent (diluted 1:10 with deionised water) and incubated for 5 min in the dark at RT. Then, 750 μl of 60 g/l sodium bicarbonate solution was added and further incubated in the dark for 90 min at 30°C. The absorbance was read at 725 nm. Gallic acid was used as the standard reference (50–1600 μg/ml). The results are expressed as mg gallic acid equivalents per gram of the extract.

### Total flavonoids content

The total flavonoids content was determined using as previously described [26]. Quercetin (3.91 - 250 μg/ml in methanol) was used as a standard reference. The standard and the extracts solutions (500 μl) were mixed with 0.1 ml of 10% (w/v) aluminum chloride, 0.1 ml of 1M potassium acetate, 1.5 ml methanol and 2.8 ml water. As for the blank, both potassium acetate and aluminum chloride were not added and their volume was replaced by water. The reaction mixture was incubated for 30 min at RT and the absorbance was taken at 415 nm.

### Statistical analysis

Statistical analysis was carried out using SPSS software version 16.0 (SPSS, Chicago, Illinois). All values are shown as a mean ± SD. Comparisons among multiple groups were done via analysis of variance (ANOVA), *P* < 0.05 was considered significant.

## Results

### Rat aortic ring assay

**Figure 1:**
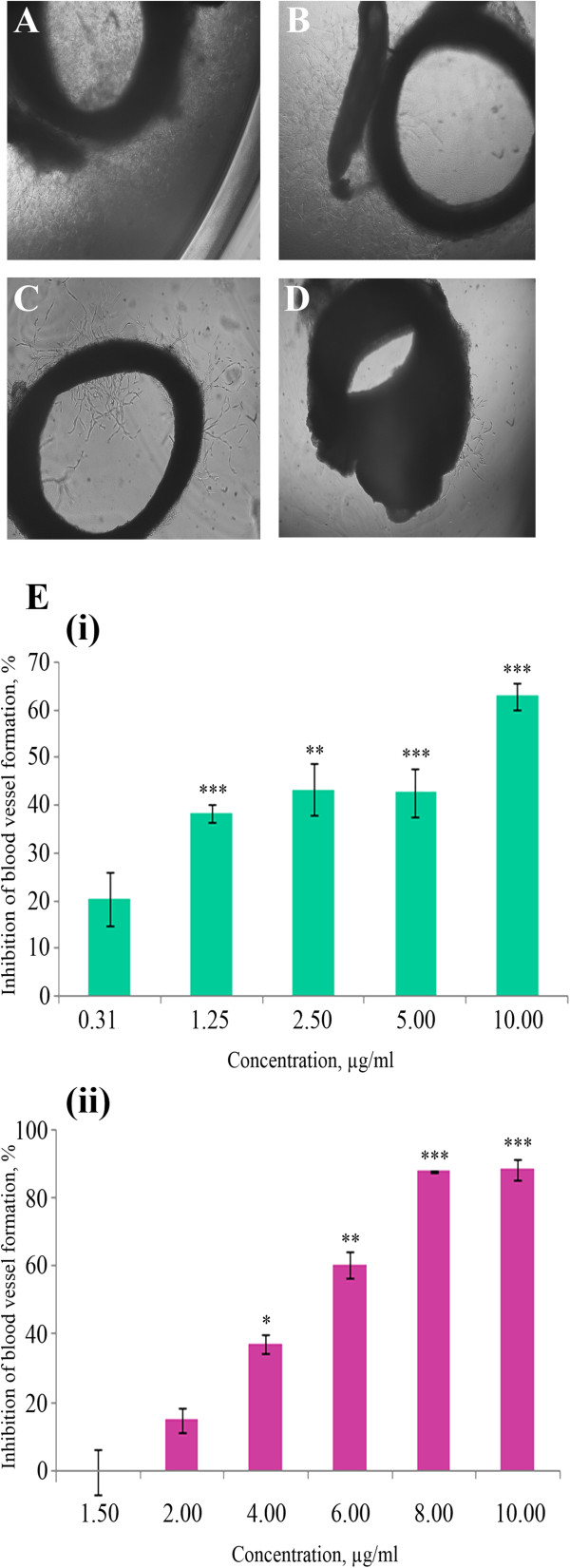
**Effect of *P. jiringa *fruit rinds extracts on microvessels outgrowth in rat aortic rings. **The rings were treated with (**A**) 1% DMSO, (**B**) 10 μg/ml EtOH extract, (**C**) 10 μg/ml EW extract and (**D**) 100 μg/ml suramin as a positive control. (**E**) Dose dependent relationship of the (i) EtOH and (ii) EW extracts on the microvessels outgrowth of the explants. The data was represented as means of three experiments (n=3), error bars equal ± SD. *P < 0.05, ** *P* <0.01 and *** *P* < 0.001.

The antiangiogenic effect of EtOH and EW extracts was first investigated using the rat aortic ring model. Figure 1A show the microvessels outgrowth from the untreated aortic rings. Figures 1B, 1C and 1D show reduction in the microvessels outgrowth by both extracts (10 μg/ml) as well as Suramin (100 μg/ml) as a positive control. IC_50s_ were calculated from the dose response curves (Figure 1E (i) and (ii)) and were found to be 5.27 ± 0.81 μg/ml and 4.45 ± 0.63 μg/ml for EtOH and EW extracts, respectively. Suramin, as a positive control, showed almost 100% inhibition of the microvessels outgrowth at 100 μg/ml.

### Effect of EtOH and EW extracts on HUVEC cells proliferation

Cytotoxic effect of both extracts was assessed by MTT test on HUVEC cells, revealing a selective cytotoxicity towards the endothelial cells with IC_50_ 0.60 ± 0.15 μg/ml (EtOH extract) and 3.01 ± 0.05 μg/ml (EW extract). The extracts’ cytotoxicity was also tested on MCF 7 (breast cancer cell line), Hep G2 (liver cancer cell line), and CCD-18Co (normal colonic fibroblasts). However, the extracts showed lower growth inhibition towards these cell lines. Table 1 depicts the calculated half maximal inhibitory concentration (IC_50s_) on all tested cell lines.

**Table 1:** **IC**_**50**_**values (μg/ml) of EtOH and EW extracts on different human cell lines**

**Extract**	**HUVEC**	**MCF 7**	**Hep G2**	**CCD-18Co**
EtOH	0.60 ± 0.15	28.66 ± 1.86	40.63 ± 0.63	4.24 ± 0.15
EW	3.01 ± 0.05	101.34 ± 1.51	38.58 ± 0.66	11.50 ± 1.02

### Inhibition of colony formation

**Figure 2:**
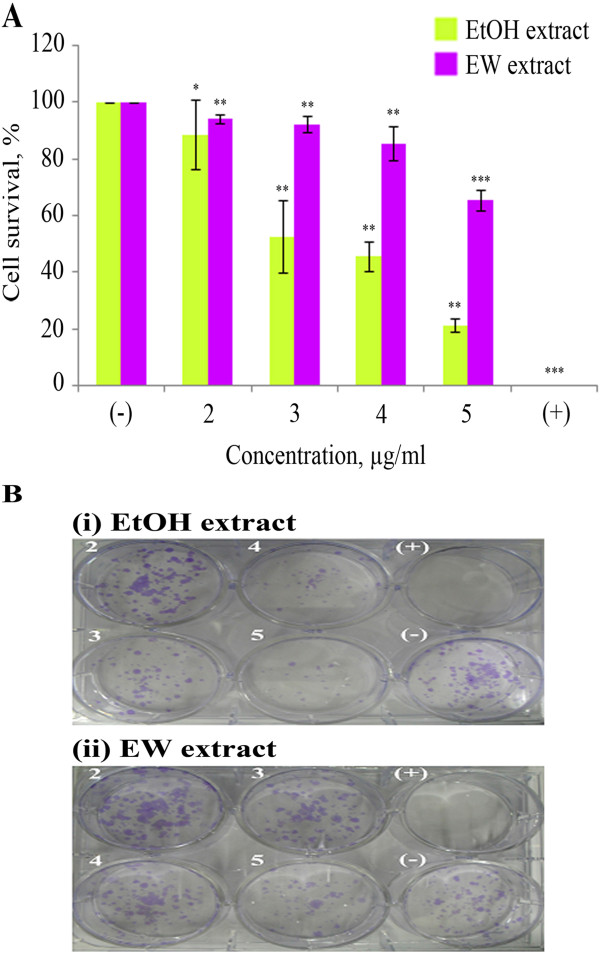
**Effect of *P. jiringa *fruit rinds extracts on clonogenicity of HUVEC cells. **(**A**) Effect of EtOH extract and EW extract. (**B**) The clonogenicity of the cells in 6-well plates after treatment with the extracts and controls. The clonogenic cell survival of HUVEC cells treated with: (−) 1% DMSO as negative control, (+) betulinic acid 20 μg/ml as positive control and indicated concentrations of the extracts (μg/ml). Values are means of two experiments, error bars equal ± SD. Plating efficiency was 13.7 ± 1.00%. *P < 0.05, **P < 0.01 and ***P < 0.001, comparing to control.

In order to figure out whether the extracts exhibit cytotoxic or cytostatic effect on HUVEC cells, the colony formation assay was performed. The results indicate that both extracts are cytostatic as shown by the percentage of survival cells [27]. The plating efficiency (PE) was 13.7 ± 1.00%. The IC_50s_ were 2.32 ± 0.28 μg/ml (EtOH extract) and 6.24 ± 0.54 μg/ml (EW extract). Figure 2 describes the survival percentages of HUVEC cells after treatment with the test extracts and their colonies after treatment.

### Cell migration

**Figure 3:**
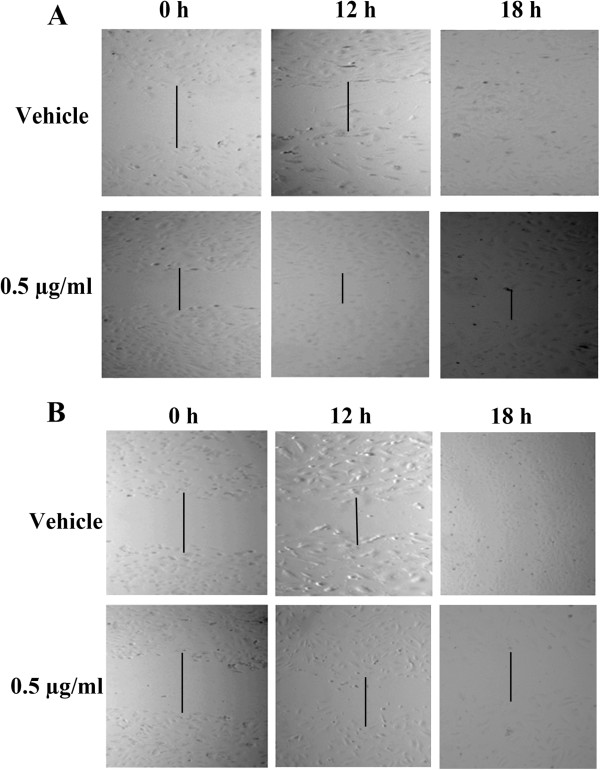
**Effect of *P. jiringa *fruit rinds extracts on HUVEC cells migration. **Upon creation of the wound, the cells were treated with 0.5 μg/ml of the extracts and 1% DMSO (vehicle) for 12 and 18 h. (**A**) EtOH extract and (**B**) EW extract.

To understand the effect of EW and EtOH extracts on HUVEC cells migration, which is one of the fundamental tools in many pathological aspects, the scratch wound healing assay was performed. Upon scratching the HUVEC cells monolayer, activated motile endothelial cells move randomly in direction of the angiogenic factors across a horizontal surface. In the present study, the results demonstrated effective reduction in the migratory capacity of the extracts-treated cells as compared to their untreated counterparts (Figure 3). In EW extract-treated cells at 0.5 μg/ml, the percentage of wound closure at 12 and 18 h was 48.02 ± 1.94% (*P* = 0.002) and 72.90 ± 0.9%, respectively. At the same concentration of the EtOH extract, the percentage of wound closure was 41.13 ± 4.38% and 49.50 ± 1.39% at 12 and 18 h, respectively.

### Tube formation

**Figure 4:**
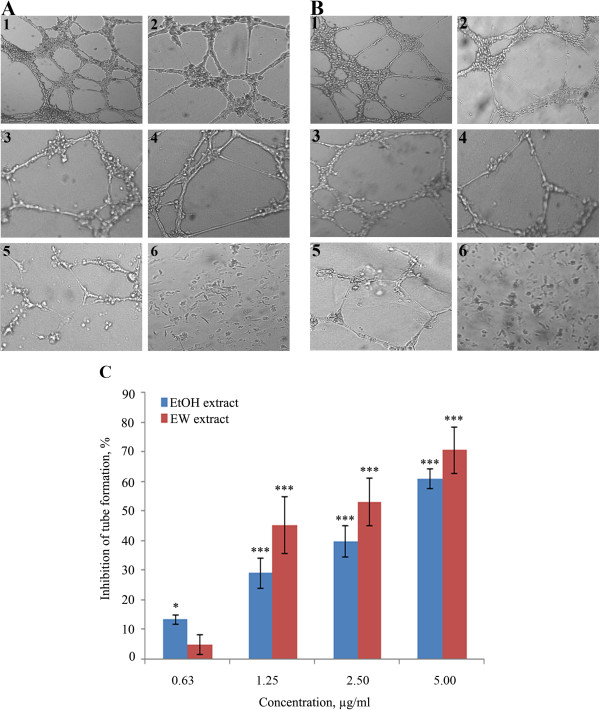
**Effect of *P. jiringa *fruit rinds extracts on tube-like formation in HUVEC cells. **(**A**) Effect of EtOH extract, (**B**) EW extract and (**C**) dose dependent relationship of the extract on the tube formation. The cells were treated with (1) 1% DMSO, (2) 0.625 μg/ml, (3) 1.25 μg/ml, (4) 2.5 μg/ml, (5) 5 μg/ml of extracts and (6) 100 μg/ml of Suramin. The dose dependent effect of the extracts is presented as mean ± SD, *P < 0.05, and ***P < 0.001.

Under normal culture conditions endothelial cells cultured on a matrigel matrix form tube-like structures within 6 h. Treatment of HUVEC cells with EtOH and EW extracts has reduced the formation of the tube-like structures in a dose dependent manner (Figure 4). The extracts showed IC_50s_ of 3.62 ± 0.83 μg/ml (EtOH) and 2.19 ± 0.19 μg/ml (EW). The positive control (suramin) inhibited endothelial tube formation by 97 ± 0.72% at 100 μg/ml.

### Inhibition of VEGF expression

The inhibitory effect of the extracts on VEGF expression was determined by quantifying VEGF 165 concentration in lysates of endothelial cells. The results showed that both extracts caused significant inhibition of VEGF expression in HUVEC cells. The VEGF concentration in cell lysates in cells treated with EtOH extract at 2.5 μg/ml (57.77 ± 4.65 pg/ml) (*P* = 0.004) and EW extract (69.06 ± 3.71 pg/ml) (*P* < 0.007) was significantly lower than that measured in untreated cells (253.36 ± 8.19 pg/ml) (*P* = 0.01). Hence, the present results propose for down-regulation of VEGF expression as one of the mechanism of actions involved.

### *In vivo* inhibition of CAM neovascularisation

**Figure 5:**
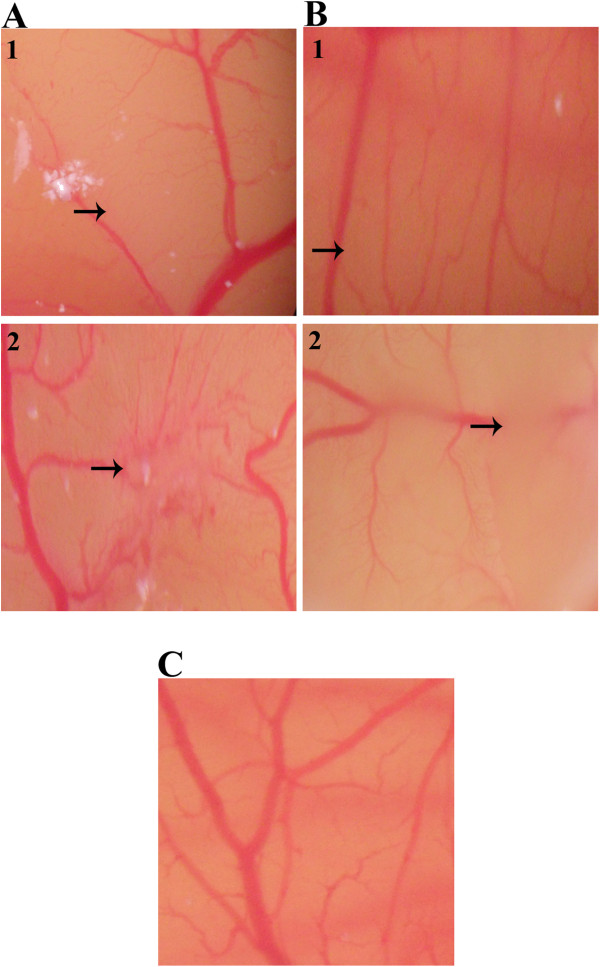
**Effects of *P jiringa *fruit rind extracts on neovascularisation in chick chorioallantoic membrane. **(**A**) CAM treated with EtOH extract and (**B**) EW extract. The CAMs were treated with (1) 25 μg (2) 50 μg extracts and (**C**) 1% EtOH.

Inhibition of normal vascularisation in chick embryo was observed by the treatment of the CAM with 25 and 50 μg of EtOH and EW extracts. Figure 5C represents normal vascularisation in the untreated CAM which consisted of primary, secondary and tertiary microvessels. In comparison, the CAM treated with 25 and 50 μg of EtOH and EW extracts displayed distorted vascularisation as well as perturbation on existing vasculatures (Figure 5A1 and 5A2; Figure 5B1 and 5B2). The percentage nhibition in EtOH extract treated CAMs was 32.75 ± 5.59% (25 μg) (*P* = 0.01) and 62.56 ± 4.20% (50 μg) while that in EW extract treatment was 51.30 ± 6.50% (25 μg) and 71.11 ± 3.08% (50 μg).

### DPPH scavenging activity

The results showed that the extracts possess potent scavenging capacity of the stable free radical DPPH. EW extract was more effective free radical scavenging agent with IC_50_ 18.48 ± 1.60 μg/ml than the EtOH extract which showed an IC_50_ of 33.52 ± 2.05 μg/ml.

### Phytochemical analysis

The preliminary phytochemical analysis revealed the presence of phenolics, flavonoids, terpenoids and alkaloids in both extracts. The TLC plates which were sprayed with the natural product reagent and enhanced with 5% ethanolic PEG 4000’ showed presence of different flavonoid compounds. Appearance of blue bands indicates the presence of phenolic acids; yellow-orange bands indicate the presence of flavonols and yellow-green bands indicate flavones.

Spraying with Folin-Ciocalteu reagent and enhancing with 20% sodium carbonate solution has marked the presence of phenolic compounds in all extracts, indicated by appearance of dark blue bands. Spraying the TLC plates with anisaldehyde reagent indicate the presence terpenoids in both extracts, as indicated by the deep purple bands, however with higher band intensity in EtOH extract than in EW extract. Presence of alkaloids was also noted in both extracts, as shown by the presence of brown bands upon spraying with Dragendorff reagent. From these results, it can be seen that *P. jiringa* extracts contain abundant number of phyto-constituents which may contribute to various pharmacological activities.

### Total phenolics and total flavonoids

The EtOH and EW extracts were subjected to a quantitative analysis of total phenolics and flavonoids. In general, EW and EtOH extracts has showed relatively similar amount of total phenolics and flavonoids. EtOH extract contained 143.95 ± 0.22 mg/g total phenolics and 2.21 ± 0.17 mg/g of total flavonoids while EW extract contained 146.22 ± 1.17 mg/g total phenolics and 2.84 ± 0.83 mg/g total flavonoids.

## Discussion

Plants contain tremendous amount of phytochemical constituents such as phenolics and flavonoids compounds, these have a great potential in promoting and maintaining a good health [28]. Antioxidants from plant origin have always been tagged with possibilities in treating and lowering the risk of various diseases such as inflammation and cancer [4]. Antioxidants have the ability to scavenge reactive oxygen species that may cause damage to DNA, proteins and lipids. In addition, antioxidants may suppress cancer cells through affecting cyclooxygenase-2 enzyme or inhibiting oncogene expression [28].

Phytochemical analysis of both EtOH and EW extracts of *P. jiringa* fruit rinds revealed the presence of various secondary metabolites including phenolics, flavonoids, terpenoids and alkaloids. In support of the present finding, previous GC-MS-TOF analysis of *P. jiringa* seeds extracts prepared by carbon dioxide supercritical extraction has shown the presence of flavonoids, terpenoids, alkaloids, vitamin E, allyl sulphur and some fatty acids [13,14].

EW extract showed higher potency in scavenging the DPPH free radicals than EtOH extract. Significant relationship between elevated antioxidant activities with high amount of total phenolics content has been extensively discussed [29-31]. Generally, the antioxidant activity of phenolics is greatly contributed by their structures, stressing on the presence of hydrogen-donating hydroxyl groups, and those with more hydroxyl groups possess greater antioxidant capacity [29]. Several studies have shown anti-mutagenic and anti-carcinogenesis effects of phenolic acids such as chlorogenic and caffeic acids due to their high antioxidant effect [31]. Previously, methanolic extract of *P. jiringa* seeds was found to inhibit Epstein-Barr Virus (EBV) activation in Raji cells, a model of anti-tumour screening, indicating the potential anti-cancer effect of *P. jiringa*[32]. The present study found relatively high concentration of total phenolics in both extracts and potent antioxidant activity which suggests a possible chemopreventive effect.

Angiogenesis is essential in tumour growth and metastasis as the process provides necessary oxygen and nutrition for the growing tumour [2]. Our results showed that both EtOH and EW extracts potently inhibited the outgrowth of new blood vessels in the rat aortic rings in a dose-dependent manner. At the same time the extracts showed selective anti-proliferative effect towards the endothelial cells compared to other human cancer cell lines. The colony formation assay was then performed in order to make distinction between cytotoxic and cytostatic response. The results indicate that EtOH and EW extracts have cytostatic effect on HUVEC cells as shown by gradual decrease in the cell survival percentage after removal of the extracts. Hence, their antiangiogenic effect may be explained due to the cytostatic effect on HUVEC cells particularly at low concentration of the extracts.

Collectively, the anti-proliferation and rat aortic ring assay results indicate that the antiangiogenic effect of the extracts is due to the selective inhibition of the growth of the endothelial cells. During degradation of the vascular basement membrane and extracellular matrix, activated endothelial cells migrate into the perivascular space, differentiate and form capillary network [33]. The significant inhibition of endothelial cells migration at low extract concentration (0.5 μg/ml) and the inhibition differentiation of endothelial cells on matrigel indicate these steps in the angiogenesis cascade as possible targets of the *P. jiringa* active principles*.*

In order to get deeper insights into the mechanism of the antiangiogenic activity of the extracts, the effect on the VEGF expression in endothelial cells was investigated. Vascular endothelial growth factor (VEGF) plays a crucial role in regulation of angiogenesis such as proliferation, migration and survival of endothelial cells. The results showed that VEGF expression has been significantly reduced in both extracts at subcytotoxic concentration of 2.5 μg/ml (*P* < 0.01), proposing for down-regulation of VEGF expression as the mechanism of action involved in inhibiting the angiogenesis cascade. Overexpression of VEGF contributes to cancer growth and metastasis [34], hence *P. jiringa* extracts may provide a new source of VEGF inhibitors as anti-tumour candidates.

In order to confirm that the antiangiogenic effect of *P. jiringa* extracts is reproduced *in vivo*, CAM assay was conducted. Treatment of the CAMs with either the EtOH or EW extracts changed the vascularisation pattern; both extracts inhibited the new blood vessels formation in the treated CAMs as well as distortion of existing vasculature. This result further supports the antiangiogenic activity of *P. jiringa.*

## Conclusions

The present study reports for the first time the inhibition of angiogenesis by *P. jiringa* extracts by blocking the VEGF expression leading to inhibition of endothelial cell proliferation, migration, and differentiation into a functional capillary network on matrigel matrix. This plant may provide a new source of antiangiogenesis agents which can be considered as potential candidates in the treatment of angiogenesis related diseases such as cancer, psoriasis, rheumatoid arthritis and diabetic retinopathy.

## Competing interests

The authors declare that they have no competing interests.

## Authors’ contributions

NSM conducted the plant extraction, cell culture work, rat aortic ring assay, antioxidant assays, performed the statistical analysis and wrote the manuscript. ZDN conducted the tube formation assay, participated in designing the experimental details and critically revising the paper. AFAA conducted the *in vivo* CAM assay and critically revised the paper. AS conducted the thin layer chromatography and interpreted the results and revised the work. NI conducted the rat aortic ring assay and cell culture work. AMSAM participated in designing and interpreting the work, and wrote and revised the paper. ZI participated in designing and interpreting the phytochemistry work. All authors read, edited and approved the final manuscript.

## Pre-publication history

The pre-publication history for this paper can be accessed here:

http://www.biomedcentral.com/1472-6882/12/210/prepub

## References

[B1] Folkman J What is the evidence that tumors are angiogenesis dependent? J Natl Cancer Inst 1990 82 1 4 10.1093/jnci/82.1.4 1688381

[B2] Folkman J Tumor Angiogenesis: Therapeutic Implications N Engl J Med 1971 285 21 1182 1186 10.1056/NEJM197111182852108 4938153

[B3] Folkman J Angiogenesis and apoptosis Semin Cancer Biol 2003 13 2 159 167 10.1016/S1044-579X(02)00133-5 12654259

[B4] Fernández-Pachón M Villano D García-Parrilla M Troncoso A Antioxidant activity of wines and relation with their polyphenolic composition Anal Chim Acta 2004 513 1 113 118 10.1016/j.aca.2004.02.028

[B5] Hc-C C Lo Y-J Lu F-J Xanthine Oxidase Inhibitors from the Leaves of Alsophila Spinulosa (HOOK) Tryon J Enzyme Inhib Med Chem 1994 8 1 61 71 10.3109/14756369409040777 7539070

[B6] Cos P Ying L Calomme M Hu JP Cimanga K Van Poel B Pieters L Vlietinck AJ Berghe DV Structure-Activity Relationship and Classification of Flavonoids as Inhibitors of Xanthine Oxidase and Superoxide Scavengers J Nat Prod 1998 61 1 71 76 10.1021/np970237h 9461655

[B7] Cao Y Cao R Angiogenesis inhibited by drinking tea Nature 1999 398 6726 381 381 10.1038/18793 10201368

[B8] Lamy S Blanchette M Michaud-Levesque J Lafleur R Durocher Y Moghrabi A Barrette S Gingras D Béliveau R Delphinidin, a dietary anthocyanidin, inhibits vascular endothelial growth factor receptor-2 phosphorylation Carcinogenesis 2006 27 5 989 16308314 10.1093/carcin/bgi279

[B9] Sartippour MR Shao ZM Heber D Beatty P Zhang L Liu C Ellis L Liu W Go VL Brooks MN Green tea inhibits vascular endothelial growth factor (VEGF) induction in human breast cancer cells J Nutr 2002 132 8 2307 12163680 10.1093/jn/132.8.2307

[B10] Lamy S Gingras D Béliveau R Green tea catechins inhibit vascular endothelial growth factor receptor phosphorylation Cancer Res 2002 62 2 381 11809684

[B11] Barceloux DG Djenkol Bean [Archidendron jiringa (Jack) I. C. Nielsen] Medical Toxicology of Natural Substances: Foods, Fungi, Medicinal Herbs, Toxic Plants, and Venomous Animals 2008 John Wiley & Sons, Inc, Hoboken, New Jersey 59 61

[B12] Zakaria M Ali Mohd M Traditional Malay Medicinal Plants 2010 Institut Terjemahan Negara Malaysia Berhad, Kuala Lumpur

[B13] Mohd Azizi CY Nik Norulaini NA Wahyu BS Mohd Omar AK Supercritical carbon dioxide extraction of constituents of pithecellobium jiringan seeds and their identification using time of flight gas spectrometry 2006

[B14] Norulaini NANIK Zaidul ISM Azizi CYM Zhari I Noramin MN Sahena F Omar AKM Supercritical carbon dioxide fractionation of pithecellobium jiringan jack seed compositions using fast gas chromatography time of flight mass spectrometry J Food Process Eng 2010 34 5 1746 1758

[B15] Brown KJ Maynes SF Bezos A Maguire DJ Ford MD Parish CR A novel in vitro assay for human angiogenesis Lab Investig 1996 75 4 539 555 8874385

[B16] Nicosia RF Lin YJ Hazelton D Qian X Endogenous regulation of angiogenesis in the rat aorta model. Role of vascular endothelial growth factor Am J Pathol 1997 151 5 1379 5 9358764 PMC1858079

[B17] Mosmann T Rapid colorimetric assay for cellular growth and survival: Application to proliferation and cytotoxicity assays J Immunol Methods 1983 65 1–2 55 63 6606682 10.1016/0022-1759(83)90303-4

[B18] Franken NAP Rodermond HM Stap J Haveman J van Bree C Clonogenic assay of cells in vitro Nat Protocols 2006 1 5 2315 2319 10.1038/nprot.2006.339 17406473

[B19] Liang CC Park AY Guan JL In vitro scratch assay: a convenient and inexpensive method for analysis of cell migration in vitro Nat Protoc 2007 2 2 329 333 10.1038/nprot.2007.30 17406593

[B20] Bandyopadhyay A YONG Z Malik SN Kreisberg J Brattain MG Sprague EA JIAN L Lopez-Casillas F Sun LZ Extracellular domain of TGFÎ2 type III receptor inhibits angiogenesis and tumor growth in human cancer cells Oncogene 2002 21 22 3541 3551 10.1038/sj.onc.1205439 12032856

[B21] West DC Thompson WD Sells PG Burbridge MF Angiogenesis assays using chick chorioallantoic membrane Methods Mol Med 2001 46 107 130 21340916 10.1385/1-59259-143-4:107

[B22] Nassar ZD Aisha AFA Ahamed MBK Ismail Z Abu-Salah KM Alrokayan SA Majid AMSA Antiangiogenic properties of Koetjapic acid, a natural triterpene isolated from Sandoricum koetjaoe Merr Cancer Cell International 2011 11 1 12 10.1186/1475-2867-11-12 21524294 PMC3111336

[B23] Sharma OP Bhat TK DPPH antioxidant assay revisited Food Chem 2009 113 4 1202 1205 10.1016/j.foodchem.2008.08.008

[B24] Trease G Evans W Pharmacognosy, 12* edition English Language Book Society/Bailliere Tindall 1983 1

[B25] Lizcano LJ Bakkali F Begoña Ruiz-Larrea M Ignacio Ruiz-Sanz J Antioxidant activity and polyphenol content of aqueous extracts from Colombian Amazonian plants with medicinal use Food Chem 2010 119 4 1566 1570 10.1016/j.foodchem.2009.09.043

[B26] Kosalec I Bakmaz M Pepeljnjak S Vladimir-Knezevic S Quantitative analysis of the flavonoids in raw propolis from northern Croatia Acta Pharm 2004 54 1 65 72 15050046

[B27] Houghton P Howes MJ Lee C Steventon G Uses and abuses of in vitro tests in ethnopharmacology: visualizing an elephant J Ethnopharmacol 2007 110 3 391 400 10.1016/j.jep.2007.01.032 17317057

[B28] Olsson ME Gustavsson KE Andersson S Nilsson Ã Duan RD Inhibition of cancer cell proliferation in vitro by fruit and berry extracts and correlations with antioxidant levels J Agric Food Chem 2004 52 24 7264 7271 10.1021/jf030479p 15563205

[B29] Cai Y Luo Q Sun M Corke H Antioxidant activity and phenolic compounds of 112 traditional Chinese medicinal plants associated with anticancer Life Sci 2004 74 17 2157 2184 10.1016/j.lfs.2003.09.047 14969719 PMC7126989

[B30] Yang CS Landau JM Huang MT Newmark HL Inhibition of carcinogenesis by dietary polyphenolic compounds Annu Rev Nutr 2001 21 1 381 406 10.1146/annurev.nutr.21.1.381 11375442

[B31] Tapiero H Tew K Nguyen Ba G Mathe G Polyphenols: do they play a role in the prevention of human pathologies? Biomed Pharmacother 2002 56 4 200 207 10.1016/S0753-3322(02)00178-6 12109813

[B32] Murakami A Ali AM Mat-Salleh K Koshimizu K Ohigashi H Screening for the in vitro anti-tumor-promoting activities of edible plants from Malaysia Biosci Biotechnol Biochem 2000 64 1 9 16 10.1271/bbb.64.9 10705442

[B33] Eccles SA Court W Patterson L Sanderson S Martin S, Murray C In Vitro Assays for Endothelial Cell Functions Related to Angiogenesis: Proliferation, Motility, Tubular Differentiation, and Proteolysis Methods in Molecular Biology, Angiogenesis Protocols 2009 467 Humana Press, Clifton, New Jersey 159 181 10.1007/978-1-59745-241-0_9 19301670

[B34] Kondo T Ohta T Igura K Hara Y Kaji K Tea catechins inhibit angiogenesis in vitro, measured by human endothelial cell growth, migration and tube formation, through inhibition of VEGF receptor binding Cancer Lett 2002 180 2 139 144 10.1016/S0304-3835(02)00007-1 12175544

